# MicroRNA-124-3p Attenuated Retinal Neovascularization in Oxygen-Induced Retinopathy Mice by Inhibiting the Dysfunction of Retinal Neuroglial Cells through STAT3 Pathway

**DOI:** 10.3390/ijms241411767

**Published:** 2023-07-21

**Authors:** Yiwen Hong, Yishen Wang, Yamei Cui, Jianying Pan, Shudi Mao, Yanjie Zhu, Tao Wen, Tianyuan Qi, Aoxiang Wang, Yan Luo

**Affiliations:** State Key Laboratory of Ophthalmology, Zhongshan Ophthalmic Center, Sun Yat-Sen University, Guangzhou 510060, China

**Keywords:** miR-124-3p, pathological retinal neovascularization, hypoxia, inflammatory response, necroptosis, STAT3

## Abstract

MicroRNA (miRNA) is a non-coding RNA that can regulate the expression of many target genes, and it is widely involved in various important physiological activities. MiR-124-3p was found to associate with the normal development of retinal vessels in our previous study, but the mechanism of its anti-angiogenic effect on pathological retinal neovascularization still needed to be explored. Therefore, this study aimed to investigate the effect and mechanism of miR-124-3p on retinal neovascularization in mice with oxygen-induced retinopathy (OIR). Here, we found that intravitreal injection of miR-124-3p agomir attenuated pathological retinal neovascularization in OIR mice. Moreover, miR-124-3p preserved the astrocytic template, inhibited reactive gliosis, and reduced the inflammatory response as well as necroptosis. Furthermore, miR-124-3p inhibited the signal transducer and activator of transcription 3 (STAT3) pathway and decreased the expression of hypoxia-inducible factor-1α and vascular endothelial growth factor. Taken together, our results revealed that miR-124-3p inhibited retinal neovascularization and neuroglial dysfunction by targeting STAT3 in OIR mice.

## 1. Introduction

Retinal neovascularization is one of the primary causes of blindness in various ocular vascular diseases, including retinopathy of prematurity (ROP), age-related macular degeneration, and proliferative diabetic retinopathy. For example, ROP is one of the main causes of infantile blindness. Approximately 65.8% of infants weighing less than 1251 g and 81.6% of infants weighing less than 1000 g at birth develop ROP [1]. As retinal vascularization is incomplete in premature infants, high oxygen therapy leads to the cessation of normal vessel formation and causes pathological retinal neovascularization (RNV) [2]. Treatments for repressing RNV have evolved from photocoagulation destruction to anti-vascular endothelial growth factor (VEGF) agents [2,3,4]. Although anti-VEGF therapy is widely used to suppress RNV, there are some concerns regarding its adverse effects, including long-term ocular and systemic developmental consequences, in addition to the requirement for frequent injections and the possibility of an incomplete response [5]. Hence, it is essential to explore more effective and safer therapies for pathological RNV.

Besides the vascular endothelial cells in the retina, it is widely recognized that retinal glial cells including macroglia and microglia also play important roles in RNV [6,7,8,9]. Retinal macroglia such as astrocytes and Müller cells take part in retinal development, function maintenance, and metabolism. They can secrete VEGF to guide the angiogenic sprouts and the development of retinal angiogenesis, and support retinal neurons [8,10,11]. Retinal microglia, the resident immune cells, are involved in the maintenance of the retinal microenvironment [12]. Under normal circumstances, microglia are in a quiescent state and only become activated in response to inflammation or hypoxia. Retinal microglia release massive amounts of inflammatory cytokines and produce pro-angiogenic factors to promote pathological RNV development in the oxygen-induced retinopathy (OIR) mouse model [9,13]. However, a recent study has indicated that hypoxia induces necroptosis and the excess release of fibroblast growth factor 2 (FGF2) in microglia [14]. Thus, the protection of retinal glial cells should represent a novel and ideal strategy to inhibit RNV.

MicroRNA (miRNA) is a non-coding RNA that can regulate the expression of approximately 60% of coding genes at the post-transcriptional level in the human genome. It is widely involved in the regulation of various physiological and pathological activities, such as development and metabolism [15]. Recently, several effective miRNA antagonists and mimics, and their delivery media have been developed, providing a promising prospect for miRNAs in the treatment of ocular neovascular diseases [16]. Our previous studies have shown that miR-124-3p expression in mouse retinas increases after birth, and its related pathways predicted by bioinformatics analysis are associated with retinal vascular development [17,18]. MiR-124 is mainly expressed in the inner nuclear layer (INL) and outer limiting membrane (OLM) of the retina, with some low-level expression in the ganglion cell layer (GCL) in healthy human retinal tissue [19]. Studies have demonstrated that miR-124-3p can regulate the activation of microglia in the central nervous system and suppress retinal inflammation [19,20]. Taken together, these results imply that miR-124-3p might be an effective alternative treatment for RNV.

Therefore, our study aimed to investigate the effect and mechanism of miR-124-3p on pathological RNV and retinal glial cells using the OIR mouse model.

## 2. Results

### 2.1. The Expression of MiR-124-3p Decreased in P12 OIR Mouse Retinas

**Figure 1:**
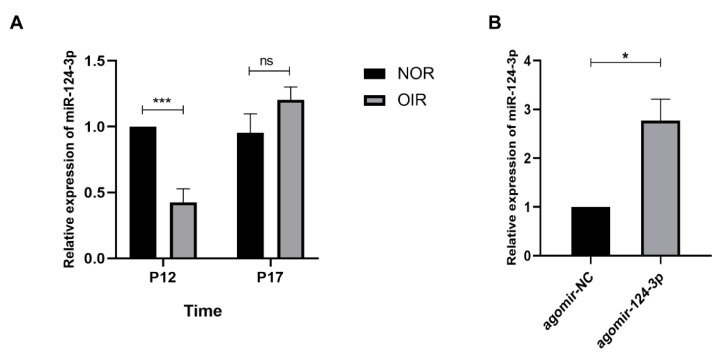
Expression of miR-124-3p in the OIR mouse retinas. (**A**) The expression of miR-124-3p in the retinas of normal mice and OIR mice at P12 and P17 (n=6). (**B**) The expression of miR-124-3p in the retinas of P17 OIR mice after intravitreal injection of agomir-124-3p or agomir-NC at P12. (n=4). The data are expressed as mean ± SD. NOR, normal group.∗p<0.05; ∗∗∗p<0.001;  ns, not significant.

To identify the change in miR-124-3p expression in the OIR mice, we performed RT-qPCR at postnatal day 12 (P12) and P17 in OIR mice. The miR-124-3p expression in the OIR mice was significantly decreased compared with the normal mice at P12, while there was no significant difference between normal mice and OIR mice at P17 (Figure 1A). Hence, OIR mice at P12 were injected intravitreally with agomir-124-3p or the agomir negative control (agomir-NC) to investigate whether the overexpression of miR-124-3p attenuated pathological RNV in OIR mice. The miR-124-3p expression was significantly increased at P17 in the agomiR-124-3p group compared with the agomir-NC group after the intravitreal injection at P12 (Figure 1B).

### 2.2. MiR-124-3p Alleviated Retinal Neovascularization in the OIR Mouse Retinas

**Figure 2:**
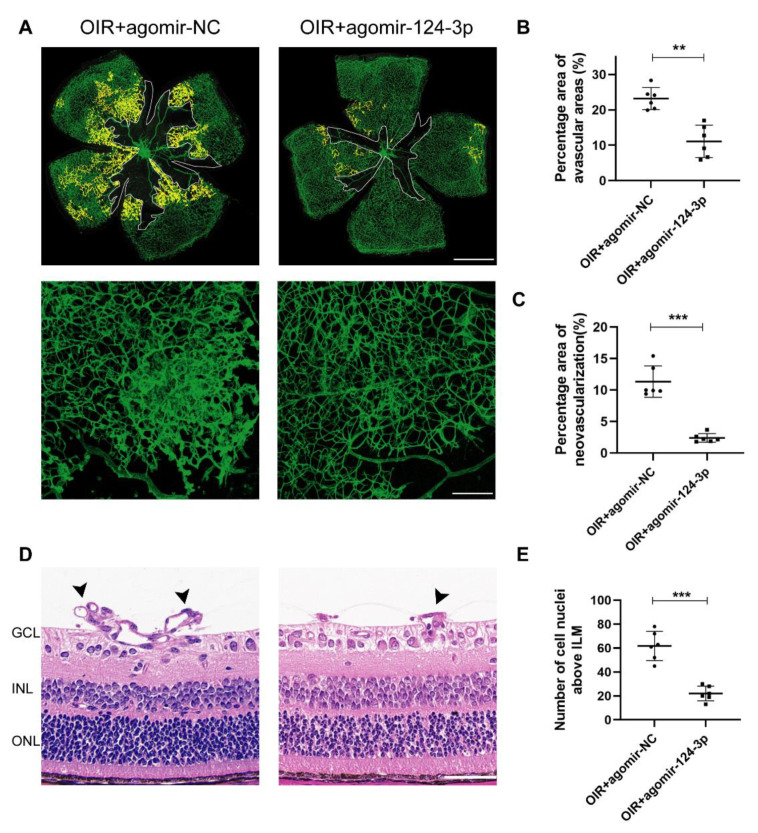
The effect of miR-124-3p on avascular areas and neovascularized areas in OIR mouse retinas. (**A**, upper image) Retinas of OIR mice from the OIR+agomir-NC group and OIR+agomir-124-3p group were harvested at P17 and subjected to whole-mount immunostaining with IB4 (green), indicating neovascular areas (filled with yellow) and avascular areas (outlined with white). Scale bar, 1000 µm. (**A**, lower image) The higher-magnification images from each group were presented. Scale bar, 200 µm. (**B**) Avascular areas and (**C**) neovascularized areas were qualified (n=6). (**D**) Neovascular cell nuclei above the internal limiting membrane (ILM) represented extent of retinal neovascularization. The arrows denote neovascular tufts. Scale bar, 50 µm. (**E**) Statistical analysis of the number of neovascular cell nuclei above the ILM in OIR+agomir-NC group and OIR+agomir-124-3p group at P17 (n=6). The data are expressed as mean ± SD. GCL, ganglion cell layer; INL, inner nuclear layer; ONL, outer nuclear layer. ∗∗p<0.01; ∗∗∗p<0.001.

To evaluate the retinal vessels, whole-mount retinal immunofluorescence stained with IB4 was used in the OIR+agomir-NC group and OIR+agomir-124-3p group at P17 (Figure 2A, upper image), and the high-power images were observed (Figure 2A, lower image). After miR-124-3p overexpression, neovascular and avascular areas in the retinas were both dramatically reduced (Figure 2B,C). Furthermore, we performed hematoxylin and eosin (HE) staining of paraffin sections to measure the number of neovascular cell nuclei above the internal limiting membrane (ILM) at P17 (Figure 2D). The results showed that the number of cell nuclei anterior to the ILM in the retinas of the OIR+agomir-124-3p group was dramatically decreased compared with the OIR+agomir-NC group (Figure 2E). All of these results indicated that miR-124-3p alleviated pathological RNV in OIR mice.

### 2.3. MiR-124-3p Preserved the Astrocytic Morphology and Suppressed Müller Gliosis in the OIR Mouse Retinas

**Figure 3:**
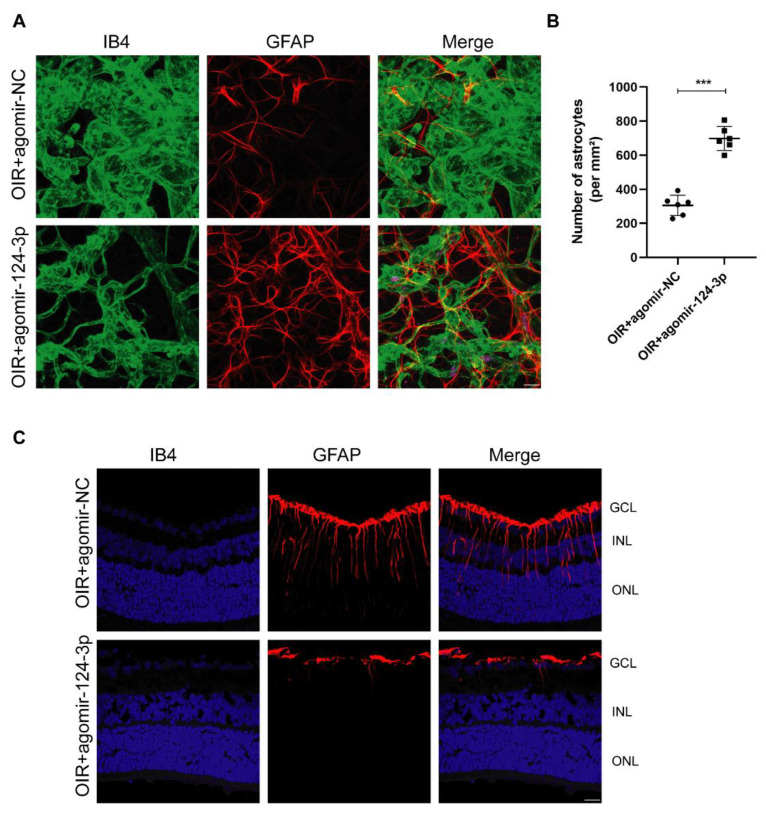
The effect of miR-124-3p on retinal macroglia (astrocytes and Müller cells) in OIR mouse retinas. (**A**) Retinas of OIR mice from the OIR+agomir-NC group and OIR+agomir-124-3p group were harvested at P17 and subjected to whole-mount immunostaining with IB4 (green) and GFAP (red). Scale bar, 20 µm. (**B**) The number of astrocytes was qualified (n=6). (**C**) Representative images of frozen sections with immunofluorescent staining with GFAP (red) in each group. The nuclei were stained with 4′,6-diamidino-2-phenylindole (DAPI) (blue). Scale bar, 20 µm. The data are expressed as mean ± SD. GCL, ganglion cell layer; INL, inner nuclear layer; ONL, outer nuclear layer. ∗∗∗p<0.001.

Macroglia in the retina, including astrocytes and Müller cells, provide the photoreceptors as well as neurons required to support neuronal activity and play important roles in eye diseases, such as RNV [21]. To investigate astrocytes in the neovascular areas in the retinas of OIR mice, we performed whole-mount immunostaining with GFAP and IB4, representing the markers for astrocytes/Müller cells and vascular cells, respectively. The results showed that after intravitreal injection, astrocytes built up a better network and retained a relatively normal distribution and morphology accompanied by decreased neovascular tufts in the OIR+agomir-124-3p group (Figure 3A,B). These results indicated that miR-124-3p preserved the astrocytic template. Next, we performed immunohistochemistry staining with GFAP on frozen sections to investigate the effect of miR-124-3p on Müller activation. GFAP is mainly expressed in retinal astrocytes under normal conditions but is expressed in Müller cells during hypoxia, such as in OIR mouse retinas [22]. We observed reactive Müller gliosis in astrocytes and Müller cells as GFAP-positive cells with stronger immunoreactivity and processes spanning the entire retina. MiR-124-3p obviously reduced the reactive expression of GFAP in astrocytes and Müller cells in OIR mouse retinas (Figure 3C). All the above results indicated that miR-124-3p not only preserved the astrocytic template but also orchestrated Müller gliosis in the retinas of OIR mice.

### 2.4. MiR-124-3p Inhibited Microgliosis and Increased M2 Microglia in the OIR Mouse Retinas

**Figure 4:**
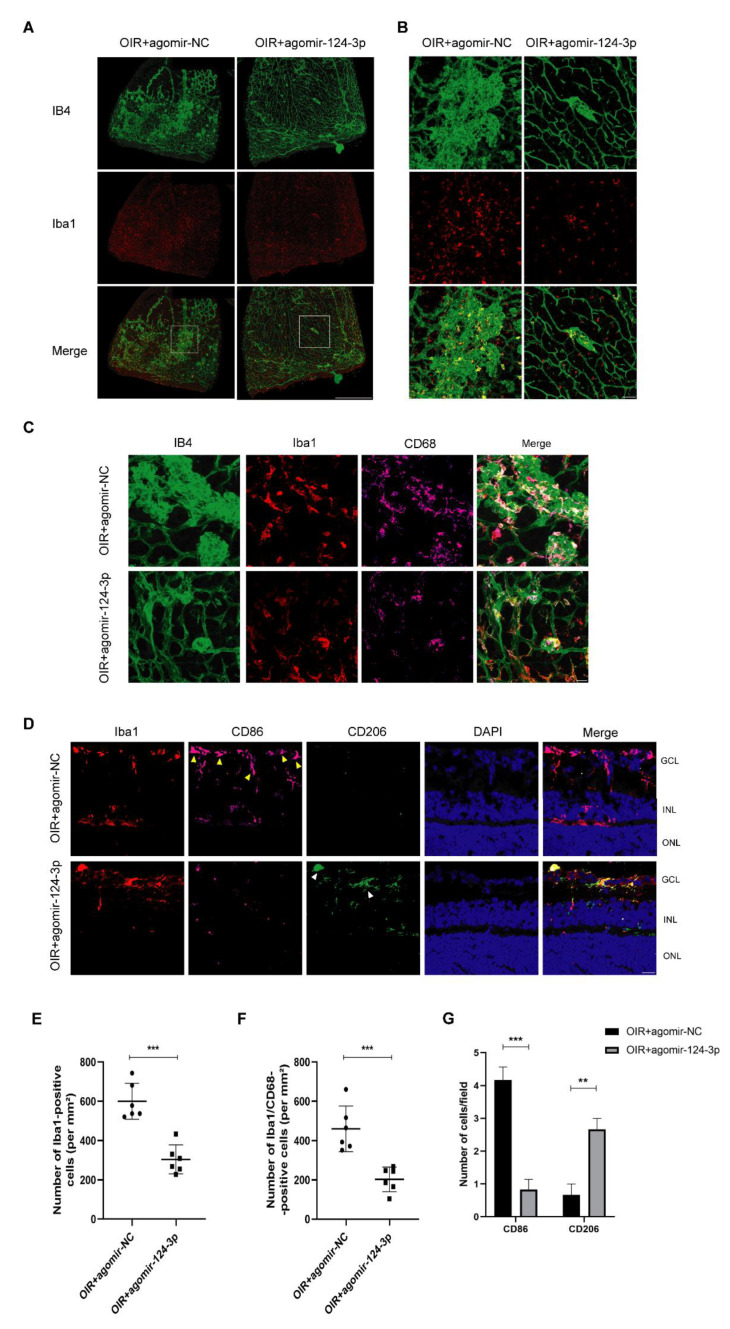
The effect of miR-124-3p on microglia in OIR mouse retinas. (**A**) Retinas of OIR mice from the OIR+agomir-NC group and OIR+agomir-124-3p group were harvested at P17 and subjected to whole-mount immunostaining with IB4 (green) and Iba1 (red). Scale bar, 200 µm. (**B**) The high-power images from the white square in (**A**) were presented. Scale bar, 50 µm. (**C**) Representative images of triple immunofluorescent staining with IB4 (green), Iba1 (red), and CD68 (violet) in each group. Scale bar, 20 µm. (**D**) Representative images of immunofluorescent staining with Iba1 (red), CD86 (violet), and CD206 (green) on retinal frozen sections. The nuclei were stained with 4ʹ,6-diamidino-2-phenylindole (DAPI) (blue). MiR-124-3p decreased the expression of CD86 (yellow arrowheads), a marker for M1 microglia, whereas the expression of CD206 (white arrowheads) increased, a marker for M2 microglia. Scale bar, 20 µm. (**E**) Quantification of the Iba1-positive cells in the neovascularized areas (n=6). (**F**) Quantification of the Iba1/CD68-positive cells in the neovascularized areas (n=6). (**G**) Quantification of M1 microglia (iba1+CD86+) and M2 microglia (iba1+CD206+) (n=6). The data are expressed as mean ± SD. GCL, ganglion cell layer; INL, inner nuclear layer; ONL, outer nuclear layer. ∗∗p<0.01; ∗∗∗p<0.001.

Next, we further investigated the effect of miR-124-3p on microglial activation, a hallmark of retinal neuroinflammation. Therefore, we focused on microglia in the neovascular areas in the retinas of the OIR mice. Immunofluorescence results indicated that Iba1-positive microglia were found to colocalize with IB4+ neovascular areas (Figure 4A), and the higher-magnification images were observed (Figure 4B). Meanwhile, immunofluorescent staining with CD68 was added, a marker for activated microglia and macrophages (Figure 4C). We found that the density of microglia and activated microglia in the neovascular area both dropped in the OIR+agomir-124-3p group (Figure 4E, F). As microglia showed a shift in functional phenotypes from M1 (pro-inflammatory) to M2 (anti-inflammatory) in the natural process of OIR mice [9], we investigated the alteration of these microglia phenotypes using immunofluorescent staining with CD86 for M1 microglia and CD206 for M2 microglia (Figure 4D). The results indicated that miR-124-3p decreased the number of M1 microglia and increased the number of M2 microglia (Figure 4G). All of the above results indicated that miR-124-3p not only orchestrated reactive microgliosis but also increased reparative microglia in the retinas of OIR mice.

### 2.5. STAT3 Is a Target Gene of MiR-124-3p in OIR Mouse Retinas

**Figure 5:**
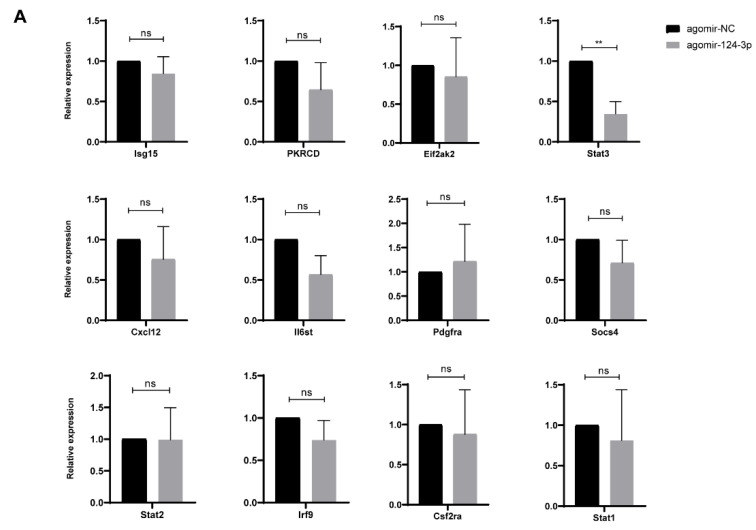

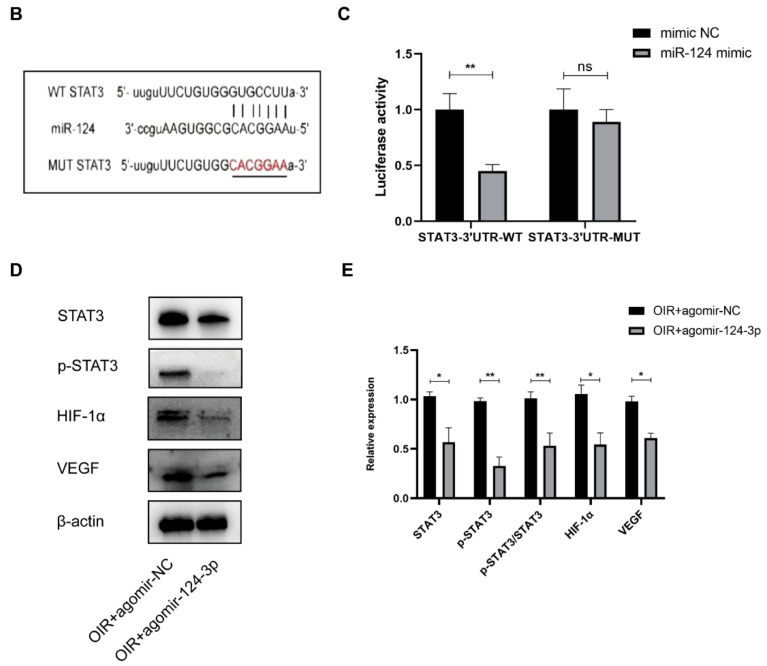
STAT3 is a target gene of miR-124-3p in OIR mouse retinas. (**A**) The mRNA expression of downstream candidate genes of miR-124-3p in each group (n=3). (**B**) The binding site of miR-124-3pwith STAT3, predicted by miRbase. (**C**) The dual-luciferase gene reporter assay verified binding of miR-124 with STAT3 (n=3). (**D**) Western blot analysis showed the expression of STAT3, p-STAT3, HIF1α, and VEGF in each group. (**E**) The histogram revealed the densitometric analysis of the average levels of STAT3, p-STAT3, HIF1α, and VEGF to β-actin, and the average levels of p-STAT3 to STAT3 (n=3). The data are expressed as mean ± SD. ∗p<0.05; ∗∗p<0.01; ns, not significant.

Our previous study determined 12 downstream candidate genes of miR-124-3p by RNA-sequencing and bioinformatics analysis in developing mouse retinas [18]. Consequently, we performed RT-PCR to validate the mRNA expression of these genes (Prkcd, Irf9, Stat3, Cxcl12, Stat1, Stat2, Isg15, Eif2ak2, Il6st, Pdgfra, Socs4, and Csf2ra) in the OIR+miR-NC group and OIR+miR-124-3p group. The results showed that the mRNA expression of STAT3 obviously decreased in the OIR+miR-124-3p group, while the expression of other genes did not show significant differences (Figure 5A). Next, we searched the binding site in the “miRbase” database and conducted a dual-luciferase gene reporter assay in HEK293T cells to determine whether STAT3 was the target gene. From the RNA sequence alignment, we found that the 3′-UTR of STAT3 mRNA included a complementary site for the seed region of miR-124-3p (Figure 5B). Meanwhile, the luciferase activities were predominantly inhibited by miR-124-3p overexpression compared with the mimic NC group in the wild-type STAT3 (WT-STAT3) group. However, there was no significant difference in the mutant-type STAT3 (MUT-STAT3) group, confirming that STAT3 is the target gene of miR-124-3p (Figure 5C). Studies have suggested that STAT3 takes part in regulating the HIF1α-VEGF pathway [23,24]. Hence, we performed a Western blot analysis to determine the protein expression (Figure 5D). Decreased levels of STAT3, p-STAT3, HIF1α, and VEGF were detected in the OIR+miR-124-3p group (Figure 5E). Meanwhile, the ratio of phosphorylated STAT3 to total STAT3 was also decreased in the OIR+miR-124-3p group. Taken together, miR-124-3p repressed the HIF1α-VEGF pathway via the targeting of STAT3 in OIR mouse retinas.

### 2.6. MiR-124-3p Attenuated Inflammation and Necroptosis in the OIR Mouse Retinas

**Figure 6:**
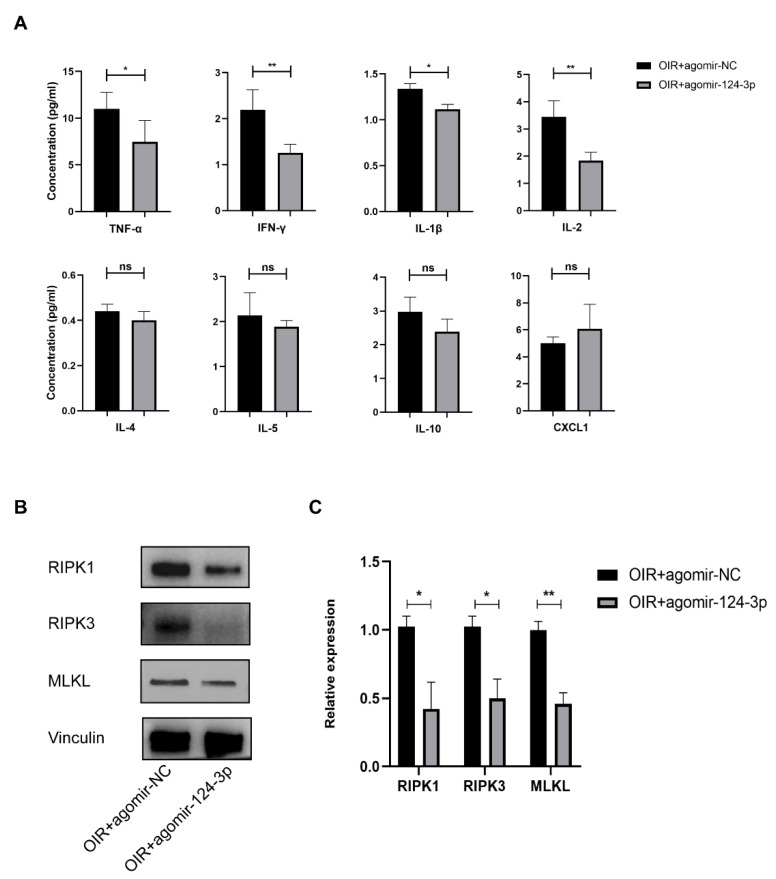
The effect of miR-124-3p on inflammation and necroptosis in OIR mouse retinas. (**A**) Luminex assay indicated the expression of eight inflammatory cytokines (TNF-α, IFN-γ, IL-1β, IL-2, IL-4, IL-5, IL-10, CXCL1) in each group (n=4). (**B**) Western blot analysis showed the expression of RIPK1, RIPK3, and MLKL in each group. (**C**) The histogram revealed the densitometric analysis of the average levels of RIPK1, RIPK3, and MLKL to Vinculin. (n=3). The data are expressed as mean ± SD. ∗p<0.05; ∗∗p<0.01;  ns, not significant.

It has been reported that the activation of the STAT3 signaling pathway is related to inflammation, leading to RNV in OIR mice [9]. Hence, we performed a Luminex assay to examine the expression of eight typical inflammatory cytokines (TNF-α, IFN-γ, IL-1β, IL-2, IL-4, IL-5, IL-10, CXCL1). We observed a prominent decrease in TNF-α, IFN-γ, IL-1β, and IL-2 protein expression from the retinas from the OIR+miR-124-3p group at P17, while no significant differences were observed in the expression of IL-4, IL-5, IL-10, and CXCL1 (Figure 6A). Necroptosis plays an important role in inflammation and was reported to affect the pathogenesis of RNV in a recent study [14,25]. Therefore, to further investigate the effect of miR-124-3p on necroptosis, a Western blot analysis was carried out to measure the expression of receptor-interacting serine/threonine protein kinase 1 (RIPK1), RIPK3, and mixed-lineage kinase domain-like protein (MLKL) (Figure 6B), the typical regulators of necroptosis. The results showed that the expression of RIPK1, RIPK3, and MLKL all significantly declined in the OIR+miR-124-3p group (Figure 6C). In summary, miR-124-3p exerted an inhibitory effect on inflammation and necroptosis in OIR mouse retinas.

## 3. Discussion

The OIR mouse is a good model for studying pathological RNV, and many miRNA therapies have been attempted on this model. The intravitreal injection of miR-18a-5p, miR-145, miR-96, and miR-181a-5 can inhibit RNV in OIR mice [26,27,28,29]. Our previous study found that miR-124-3p is one of the miRNAs that are closely related to retinal vascular development [17]. MicroRNA-124 was found to alleviate retinal vasoregression of the neurodegenerative ciliopathy-associated disease rat model and prevent rat diabetic retinopathy via regulating microglial polarization [30,31]. In this study, we found that intravitreal injection of agomir-124-3p dramatically reduced the areas of avascular and neovascularization, indicating an anti-angiogenic effect of miR-124-3p. Our study was consistent with a previous study, which found that the MALAT1/miR-124-3p/EGR1 regulatory axis is partly responsible for RNV in the OIR model [32]. The previously documented mechanism of the anti-angiogenic effect of miR-124-3p mainly focused on microglia and inflammation. In our study, we have not only shed light on them but also studied macroglia, necroptosis, and STAT3.

Vascular networks and nervous networks are two major retinal systems, so understanding the interaction among angiogenesis and the glial response in RNV is quite significant. Astrocytes and Müller cells can protect neurons, maintain the homeostasis of the retina, and take part in the control of angiogenesis [10]. Our study showed that miR-124-3p preserved the density and better morphology of astrocytes and suppressed Müller gliosis in OIR mouse retinas, which resulted in alleviating pathologic RNV. Astrocytes release VEGF to stimulate the endothelia proliferation and extension, and reduced cell number and degeneration of retinal astrocytes have been found in the OIR model [8,33,34]. Like astrocytes, Müller-cell-derived VEGF is another important contributor to RNV, and Müller cells can trigger the growth of retinal vascular endothelial cells under hypoxia [6,35]. In addition, injured Müller cells exhibit a gliotic phenotype during the hypoxic phase of OIR [36]. Persistent Müller gliosis is considered to be a poor prognosis sign, as macroglial reactive cells secrete pro-inflammatory cytokines, such as IL-1β and TNF-α, leading to retinal inflammation [37,38]. Our results suggest that the anti-inflammatory effect of miR-124-3p may contribute to inhibiting RNV.

Microglia are involved in a variety of retinal diseases, including pathological RNV, and play an important role in the inflammatory response [39]. Retinal microglia are normally quiescent, but are activated to release inflammatory cytokines to trigger auto-destructive responses in the diseased retina [39]. The number of retinal microglia around RNV tufts increase and most of them are activated in the OIR model, leading to an inflammatory response and vascular permeability [9]. In OIR mice, M1/M2 shifting of microglia is significant for the recession of RNV during the natural process [9]. Hence, treatments that focus on regulating microglial activation and polarization might be a promising option for pathological RNV. It has been demonstrated that miR-124-3p suppresses microglial activation and contributes to M2 polarization of microglia in the central nervous system [20,40]. This study confirmed that miR-124-3p also attenuated microgliosis and increased M2-polarized microglia in OIR mouse retinas. In addition, the microglia necroptosis axis has been considered as a new anti-angiogenesis therapy for pathological RNV diseases [14]. Notably, our study found that miR-124-3p inhibited necroptosis in the OIR mouse retina, which has not been documented before. Therefore, miR-124-3p had a potential anti-necroptosis effect on retinas, which could be a complementary mechanism of alleviating RNV.

It is widely recognized that STAT3 is the target gene of miR-124-3p [41,42], which was also confirmed by our dual-luciferase gene reporter assay. STAT3 is a direct transcription factor that plays important roles in inflammation, cancer, angiogenesis, and immunosuppression [43]. In the eye, STAT3 is mainly expressed in the retina, and localizes to the inner nuclear layer and ganglion cells [44,45]. Activated STAT3 predominantly localizes in the nuclei of retinal Müller (glial) cells, ganglion cells, and astrocytes but not in photoreceptors [46]. STAT3 serves as a vital regulator of retinal macroglia and microglia. The JAK/STAT3 pathway is required for the initiation of the astrogliosis-like reaction of retinal Müller cells after optic nerve injury [47]. Conditional STAT3 knockout mice eliminated Müller cell activation induced by optic nerve crush [47]. Moreover, in the injured brain and experimental glaucoma model, conditional knockout of STAT3 from astrocytes attenuated the injury-induced reactive phenotype, pro-inflammatory cytokine activation, GFAP up-regulation, and scar formation [48,49,50,51]. Meanwhile, deactivating the STAT3 pathway in cultured astrocytes in vitro can inhibit reactive astrocyte proliferation and activation [52]. As for microglia, the STAT3 signaling pathway takes part in modulating M1/M2 microglia polarization and inflammation, not only in the central nervous system but also in OIR mouse retinas [9,53,54]. JAK2 inhibition in mice with middle cerebral artery occlusion decreases STAT3 phosphorylation, thus inhibiting the expression of downstream pro-inflammatory cytokines [55]. Notably, studies have shown that STAT3 plays a crucial role in RNV [44]. In OIR mice, the M1/M2 shifting of microglia associated with the STAT3 signaling pathway is significant for the recession of RNV during the natural process [9]. In addition, STAT3 is the known upstream factor of the HIF1α-VEGF pathway, which is directly involved in the pathogenesis of pathological RNV [24,56]. Notably, our research found that the expression of STAT3, HIF1α, and VEGF reduced after miR-124-3p overexpression in OIR mice. Taken together, we drew the conclusion that miR-124-3p might alleviate RNV by inhibiting the HIF1α-VEGF pathway and inflammation via the targeting of STAT3. However, as we found that miR-124-3p had an anti-necrotic effect, this needs to be explored further as the relationship between STAT3 and necroptosis is still unclear.

## 4. Materials and Methods

### 4.1. OIR Mouse Model

C57Bl/6J mice were purchased from the Animal Research Center of the Zhongshan Ophthalmic Center (Guangzhou, China). For OIR modeling, C57BL/6J mice at P7 were exposed to 75% oxygen along with nursing mothers for 5 days and then abruptly returned to room air (21% oxygen) at P12. Mice were sacrificed at P17 for retinal isolation and analysis. All animal experiments complied with the guidelines of the Association for Research in Vision and Ophthalmology (ARVO) on the use of animals in research and were approved by the Zhongshan Ophthalmic Center Animal Care and Use Committee, Sun Yat-sen University, Guangzhou, China.

### 4.2. Intravitreal Injection

Agomirs are cholesterylated molecules that mimic endogenous mature miRNAs, while agomir-NC are random sequences of agomir molecules. Agomir-124-3p and agomir-NC (Ribobio technology, Guangzhou, China) were dissolved in normal saline at a final concentration of 0.6 nmol. The left eye was intravitreally injected with miR-124-3p agomir as the miR-124-3p overexpression group (agomir-124-3p group), and the right eye was intravitreally injected with an equal volume and concentration of agomir-NC as the control group (agomir-NC group).

Before intravitreal injection was performed, all mice at P12 were anaesthetized using isoflurane (RWD technology, Shenzhen, China). Next, 1 μL agomir-124-3p or agomir-NC was intravitreally injected using a 2.5 µL Hamilton syringe (Sigma Aldrich, Steinheim, Germany) with a 34-gauge needle under a stereoscopic microscope (Carl Zeiss, Jena, Germany). After the intravitreal injection, the needle remained in the same position for at least 20 s to prevent an outflow of the molecules and to promote distribution in the vitreous humor. Then, a topical ophthalmic antibiotic was used to prevent infection.

### 4.3. Immunofluorescence of Whole-Mount Retinas

Mice were euthanized by cervical dislocation at P17, and their eyes were enucleated and fixed with 4% paraformaldehyde (PFA) for 1 h. After removing the cornea and lens and peeling the sclera off with forceps to isolate the retina under a stereoscopic microscope, intact retinas were blocked and permeabilized in PBS containing 5% BSA and 0.5% Triton-X-100 overnight at 4 °C. Then, retinas were incubated with primary antibodies for IB4 (1:100, I21411, Invitrogen, San Diego, CA, USA), iba1 (1:200, ab178846, Abcam, Cambridge, UK), CD68 (1:100, ab53444, Abcam, Cambridge, UK), or GFAP (1:100, 80788, Cell Signaling Technology, Boston, MA, USA) overnight at 4 °C. After washing in PBS, the secondary antibodies were added and incubated for 2 h at room temperature (RT). Retinas were then washed with PBS and mounted on slides, and images were taken by confocal microscopy (LSM880, Carl Zeiss, Jena, Germany). For whole retinal mounts, six retinas were analyzed per experimental group and the extent of the neovascular or avascular areas were analyzed using ImageJ software (v1.53, National Institutes of Health, Washington, DC, USA).

### 4.4. Immunofluorescence of Retinal Frozen Section

The eyes of mice at P17 were enucleated and fixed at RT for 1 h in 4% PFA. After removing the cornea and lens, which were fixed in 4% PFA overnight, the eyes were dehydrated in 20% sucrose and embedded in OCT compound. Then, they were frozen at −20 °C overnight and cut into 10 μm thick sections. Next, the sections were washed with PBS before permeabilizing and blocking with 1% BSA and 0.25% Triton-X-100 for 1 h at RT. The sections were incubated with IB4 (1:200), iba1 (1:500), GFAP (1:200), CD86 (1:100, ab119857, Abcam, Cambridge, UK), CD206 (1:100, AF2535, R&D systems, Minneapolis, MN, USA), and VEGF (1:100, NB100-664, Novus, Centennial, CO, USA) overnight at 4 °C. After washing in PBS, sections were incubated for 2 h at RT with a mixture of secondary antibodies and DAPI. The images of retinal frozen sections were also taken by confocal microscopy. Three fields of view (at ×400 magnification) on each section and at least three sections for each eye were observed. For statistical analysis of frozen sections, six eyes were assessed for each group.

### 4.5. HE Staining

The eyeballs of mice in each group were enucleated at P17 to obtain paraffin sections. HE staining was performed, as described previously [57]. To assess the number of vitreoretinal neovascular cells nuclei, sections were photographed using a light microscope (Leica, Frankfurt, Germany). A quantitative method for HE staining is in keeping with the information detailed above for the statistical analysis of the frozen sections.

### 4.6. Protein Extraction and Western Blot Analysis

Protein was extracted from the retinas from mice in each group at P17 with RIPA (Beyotime, Shanghai, China) containing protease inhibitors. Protein concentrations were assessed by the Pierce BCA Protein Assay Kit (Thermo Scientific, San Diego, CA, USA) following the manufacturer’s instructions. Samples containing 20 μg protein were separated with sodium dodecyl sulfate-PAGE (Beyotime, Shanghai, China) and transferred to a polyvinylidene difluoride filter (PVDF) membrane (Millipore, Boston, MA, USA). Next, the PVDF membranes were blocked with 5% defatted milk in PBS–Tween 20 for 1 h at RT before incubating with diluted STAT3 antibodies (1:1000, 9139, Cell Signaling Technology, Boston, MA, USA), p-STAT3 (1:1000, 9145, Cell Signaling Technology, Boston, MA, USA), RIPK1 (1:1000, 3493, Cell Signaling Technology, Boston, MA, USA), RIPK3 (1:1000, ab62344, Abcam, Cambridge, UK), HIF-1α (1:800, ab179483, Abcam, Cambridge, UK), VEGF (1:500, NB100-664, Novus, Centennial, CO, USA), Vinculin (1:2000, 13901, Cell Signaling Technology, Boston, MA, USA), and β-actin (1:2000, 3700, Cell Signaling Technology, Boston, MA, USA) in a blocking solution overnight at 4 °C. Subsequently, the PVDF membranes were incubated with the secondary antibody HRP for 2 h at RT after washing and visualized by an enhanced chemiluminescence system (UELandy, Suzhou, China). The bands were measured using the Image Lab system (BioRad, Hercules, CA, USA).

### 4.7. RNA Extraction and qRT-PCR

RNA extraction of retinas at P17 and cDNA synthesis were performed using TRIzol reagent (Invitrogen, San Diego, CA, USA) and a cDNA synthesis kit (Takara, Kustatsu, Japan), respectively. The method of qRT-PCR was described in our previous study [18]. Information regarding the PCR primers used is summarized in Table 1. U6 and GAPDH were used as the reference genes to normalize the miRNA and total mRNA levels, respectively. Finally, the target gene expression was calculated by the relative quantitative method of 2^−ΔΔCt^.

### 4.8. Dual-Luciferase Reporter Gene Assay

The predicted binding site fragments (miR-124-3p with STAT3) and mutation fragments were inserted into the dual-luciferase reporter vector as the reporter plasmids, which were labeled as WT and MUT, respectively. Luciferase reporter vectors were co-transfected with miR-124 mimic or mimic NC (Ribobio technology, Guangzhou, China). Next, 293T cells were seeded overnight, then co-treated with miR-124-3p reagents as well as the recombinant WT/MT-vector. Renilla fluorescence served as the internal reference. The cells were collected following the protocol of a dual-luciferase kit (Beyotime, Shanghai, China). The luciferase activity was detected using a PerkinElmer EnSpire Microplate reader (Promega Corporation, Madison, WI, USA).

### 4.9. Luminex Multiplex Cytokine Assay

Protein was extracted from the retinas from mice in each group at P17 with tissue lysate (Absin, Shanghai, China) containing protease inhibitors. The production of 8 cytokines (TNF-α, IFN-γ, IL-1β, IL-2, IL-4, IL-5, IL-10, CXCL1) in retinal supernatants from each group was measured using the Mouse Cytokine Assay Kit (BIO-RAD, Richmond, VA, USA) according to the manufacturer’s instructions. The signals were detected and data analyzed by the Luminex 200 System (Thermo Scientific, San Diego, CA, USA).

### 4.10. Statistical Analysis

All data were expressed as the mean ± SD or a percentage. Data analysis was performed by Student’s t-test using the statistical software GraphPad Prism 8 (GraphPad Software, San Diego, CA, USA). The level of statistical significance in our study was p<0.05. At least three independent repeats were performed for each experiment.

## 5. Conclusions

In summary, our study found that microRNA-124-3p attenuated retinal neovascularization in OIR mice by inhibiting the dysfunction of retinal neuroglial cells through the STAT3 pathway. These results indicated that miR-124-3p might be a potential promising therapy for RNV in retinal neovascular diseases.

## Figures and Tables

**Table 1:** Primers used in quantitative real-time PCR.

miRNA and Genes	Sequencing 5′ to 3′
miR-124-3p	GCGAGGATCTGTGAATGCCAAA
U6	GCTTCGGCAGCACATATACTAAAAT
mouse Isg15 F	GGTGTCCGTGACTAACTCCAT
mouse Isg15 R	TGGAAAGGGTAAGACCGTCCT
mouse PKRCD F	CCTCCTGTACGAAATGCTCATC
mouse PKRCD R	GTTTCCTGTTACTCCCAGCCT
mouse Eif2ak2 F	ATGCACGGAGTAGCCATTACG
mouse Eif2ak2 R	TGACAATCCACCTTGTTTTCGT
mouse Cxcl12 F	TGCATCAGTGACGGTAAACCA
mouse Cxcl12 R	TTCTTCAGCCGTGCAACAATC
mouse Il6st F	CCGTGTGGTTACATCTACCCT
mouse Il6st R	CGTGGTTCTGTTGATGACAGTG
mouse Pdgfra F	TCCATGCTAGACTCAGAAGTCA
mouse Pdgfra R	TCCCGGTGGACACAATTTTTC
mouse Socs4 F	CGGAGTCGAAGTGCTGACAG
mouse Socs4 R	ACTCAATGGACGAACAGCTAAG
mouse Stat2 F	TCCTGCCAATGGACGTTCG
mouse Stat2 R	GTCCCACTGGTTCAGTTGGT
mouse Csf2ra F	CTGCTCTTCTCCACGCTACTG
mouse Csf2ra R	GAGACTCGCCGGTGTATCC
mouse Irf9 F	GCCGAGTGGTGGGTAAGAC
mouse Irf9 R	GCAAAGGCGCTGAACAAAGAG
mouse Stat3 F	CACCTTGGATTGAGAGTCAAGAC
mouse Stat3 R	AGGAATCGGCTATATTGCTGGT
mouse Stat1 F	TCACAGTGGTTCGAGCTTCAG
mouse Stat1 R	GCAAACGAGACATCATAGGCA
mouse Gapdh F	AGGTCGGTGTGAACGGATTTG
mouse Gapdh R	TGTAGACCATGTAGTTGAGGTC

## Data Availability

The datasets generated during and/or analyzed during the current study are not publicly available due to the following study request but are available from the corresponding author on reasonable request.
